# Label-Free Biomedical Imaging Using High-Speed Lock-In Pixel Sensor for Stimulated Raman Scattering

**DOI:** 10.3390/s17112581

**Published:** 2017-11-09

**Authors:** Kamel Mars, De Xing Lioe, Shoji Kawahito, Keita Yasutomi, Keiichiro Kagawa, Takahiro Yamada, Mamoru Hashimoto

**Affiliations:** 1 Research Institute of Electronics, Shizuoka University, 3-5-1 Johoku, Nakaku, Hamamatsu, Shizuoka 432-8011, Japan; kamel@idl.rie.shizuoka.ac.jp (K.M.); lioe@idl.rie.shizuoka.ac.jp (D.X.L.); kyasu@idl.rie.shizuoka.ac.jp (K.Y.); kagawa@idl.rie.shizuoka.ac.jp (K.K.); 2 Mitsubishi Electric Corporation Nagoya Works, FA System Manufacturing Department, Manufacturing Engineering Section, 1-14, Yada-minami 5-chome, Higashi-ku, Nagoya, Aichi 461-8670, Japan; Yamada.Takahiro@ah.MitsubishiElectric.co.jp; 3 Graduate School of Engineering Science, Osaka University, 1-3 Machikaneyama, Toyonaka, Osaka 560-8531, Japan; hashimoto@ist.hokudai.ac.jp; 4 Graduate School of Information Science and Technology, Hokkaido University, Kita 14, Nishi 9, Kita-ku, Sapporo 060-0814, Japan

**Keywords:** CMOS image sensor, stimulated Raman scattering, lock-in pixel, Raman shift, low-noise, high-speed modulation

## Abstract

Raman imaging eliminates the need for staining procedures, providing label-free imaging to study biological samples. Recent developments in stimulated Raman scattering (SRS) have achieved fast acquisition speed and hyperspectral imaging. However, there has been a problem of lack of detectors suitable for MHz modulation rate parallel detection, detecting multiple small SRS signals while eliminating extremely strong offset due to direct laser light. In this paper, we present a complementary metal-oxide semiconductor (CMOS) image sensor using high-speed lock-in pixels for stimulated Raman scattering that is capable of obtaining the difference of Stokes-on and Stokes-off signal at modulation frequency of 20 MHz in the pixel before reading out. The generated small SRS signal is extracted and amplified in a pixel using a high-speed and large area lateral electric field charge modulator (LEFM) employing two-step ion implantation and an in-pixel pair of low-pass filter, a sample and hold circuit and a switched capacitor integrator using a fully differential amplifier. A prototype chip is fabricated using 0.11 μm CMOS image sensor technology process. SRS spectra and images of stearic acid and 3T3-L1 samples are successfully obtained. The outcomes suggest that hyperspectral and multi-focus SRS imaging at video rate is viable after slight modifications to the pixel architecture and the acquisition system.

## 1. Introduction

Coherent Raman scattering (CRS) is gaining popularity to provide label-free biomedical imaging. The CRS finds its applications in a wide range of biology and medical imaging [1,2,3,4,5,6,7,8], in which the use of exogenous dye will perturb the molecules of interest. Another advantage of the CRS is the high sensitivity that overcomes the drawback of low-speed imaging by spontaneous Raman scattering. The modalities of CRS that has been developed and widely employed are the Coherent anti-Stokes Raman scattering (CARS) and stimulated Raman scattering (SRS). Both CARS and SRS excite the sample with a pair of lasers, namely the pump at frequency $\omega_{p}$ and the Stokes at frequency $\omega_{s}$.

**Figure 1:**
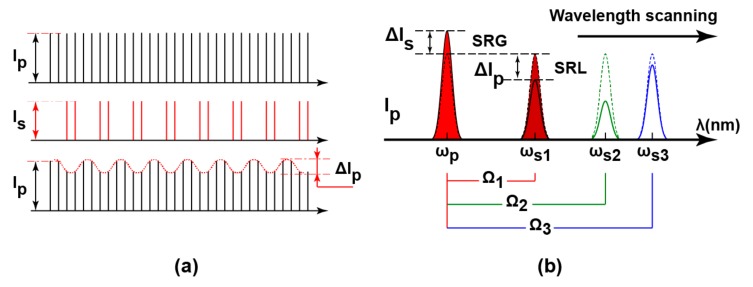
Stimulated Raman scattering (SRS): (**a**) high-speed modulation for small SRS signal; and (**b**) SRS spectrum measurement principle by wavelength scanning.

The CARS produces the anti-Stokes emission at a third frequency, $\omega_{\mathrm{as}}=2\omega_{p}-\omega_{s}$ to achieve molecular vibration. High-speed imaging using the CARS, which achieves video-rate, has been demonstrated [9]. The CARS, however, has the limitation of containing non-resonant background which distorts the Raman spectrum. Advances in CARS development have overcome the non-resonant background, with the trade-off of complicated process and analysis [10]. The SRS does not suffer from the non-resonant background and exhibits equal Raman spectrum as the spontaneous Raman scattering. In the SRS, stimulated excitation occurs when the frequency difference, $\Omega =\omega_{p}-\omega_{s}$, is at resonance with the molecular vibrational frequency. The intensity of the pump laser decreases by $\Delta I_{p}$ (stimulated Raman loss, SRL) while the intensity of the Stokes laser increases by $\Delta I_{s}$ (stimulated Raman gain, SRG) as shown in Figure 1b.

Numerous SRS systems have been implemented to gain from the advantages of SRS over CARS. The widely used methods are implemented by lock-in detection with a discrete photodiode and lock-in amplifier [11,12,13]. The recent developments of SRS realized multiplex detection based on slow modulation rate [14,15], in which at such rate the laser 1/f noise is considerably large. A customized SRS image sensor aimed to achieve multiplex detection with high modulation rate at 20 MHz has been demonstrated using a single-pixel but achieves only low signal-to-noise (SNR) ratio [16].

In this paper, we present a SRS CMOS lock-in pixel using a large area lateral electric field charge modulator (LEFM) [17]. The lock-in pixel functions as both the photodiode and lock-in amplifier, in which the modulating frequency of the lock-in pixel is synchronized with the laser system. The small SRS signal of $\Delta I_{p}$ is extracted from the large background, typically with the ratio of 10^−5^, and integrated multiples times to achieve amplification. The proposed SRS CMOS lock-in pixel works with the pump laser running at 80 MHz repetition rate and a modulation rate of 20 MHz, as shown in Figure 1a. Sinusoidal modulation is chosen over square modulation to relax the damage of the sample [12]. To validate the functionality of the lock-in pixel, the Raman spectra and images of solid state stearic acid and 3T3-L1 cells are successfully observed using the fabricated SRS CMOS lock-in pixel sensor chip. The current demonstration of the functionality of a single pixel is another important step towards the ultimate goal of realizing multi-point parallel detection with an array of pixel. In single beam irradiation method, higher peak laser power is needed to achieve high-speed imaging. Considering the photo-damage to the biological sample is mainly induced by multi-photon events, which is proportional to the peak power of the incident laser, higher laser power increases the potential of greater photo-damage to the biological samples. For single-focus and multi-focus systems obtaining signals at the same frame rate, multi-focus imaging requires longer exposure time but lesser peak laser power on each point. Such a technique reduces the induced photo-damage to the biological samples, which is highly demanded, especially for living cell imaging [18,19]. The parallel detection of SRS signals needs a customized pixel that has a function of demodulation of the modulated laser to extract weak SRS signal in huge background, and high modulation/demodulation frequency to reduce the influence of the substantially-large laser 1/f noise. The proposed 2-D CMOS lock-in pixel array that works at 20 MHz modulation frequency and has the SRS signal extraction in the huge offset fills in the need of pixel array for SRS imaging.

The rest of this paper is organized as follows: Section 2 introduces the SRS lock-in pixel and readout circuit architecture. Section 3 describes the implementation of the SRS sensor chip, the experimental setup, and the measurement results and discussions. Section 4 discusses the outlook of the implemented pixel. The final section concludes the paper.

## 2. SRS Lock-In Pixel Design

To achieve a high-speed modulation, the detector in our previous implementation [16] uses 10 × 10 sub-pixels of a pinned photodiode-based photo-charge modulator commonly known as lateral electric field charge modulator (i.e., LEFM) where all similar outputs and gates controlling signals are connected together in parallel. The small LEFM detector can ensure a very fast charge transfer of less than one nanosecond, which makes it very suitable for high-speed demodulation of several megahertz. On the other hand, the combination of such small structure to make large area detector is not very effective since not all the incident photons are captured due to the existing gap between the sub-apertures. Moreover, if the laser beam spot is smaller than the total detector area, not all the beam spot is captured in sub-apertures and this will lead to extra noise components if there is a fluctuation of the laser beam causing variation the light intensity. A new design of the photo-detector solves this matter.

### 2.1. Large Area Demodulator Design

**Figure 2:**
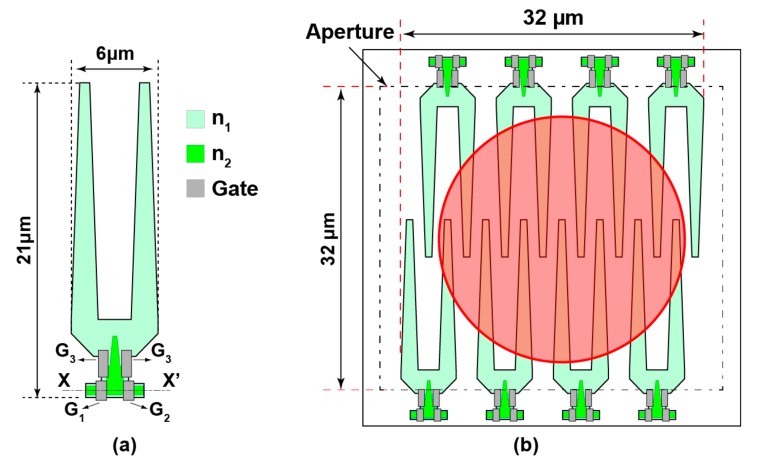
Two-step ion implantation large area Lateral electric field charge modulator (LEFM): (**a**) top view of LEFM sub-unit structure; and (**b**) top view of large area detector using eight combinations of LEFM with laser beam illustration.

To address the limitation in noise caused by the use of sub-pixels array to achieve a large area detector to obtain a better output signal stability and to improve the signal-to-noise ratio (SNR), we propose a new structure of pinned-photodiode-based detector. Figure 2a illustrates the new LEFM sub-structure with two taps of modulating gates employed to build a large area with single aperture photo-detector as depicted in Figure 2b. A combination of eight sub-structures is utilized where the incident laser beam spot is illustrated as a red circle. Such combination allows the design of a large aperture area offering the advantage that all the incident photons to this aperture are collected in contrast to the structure using 10 × 10 sub-pixel array where the sensitive area is limited to the sub-aperture area. The layout and dimension of the LEFM sub-structure are carefully designed to create an electric field slope toward the direction of the modulating gates at the bottom of the two-finger structure. Two-step ion implantation is used where the n-type layer n_2_ have a higher doping concentration than the n-type layer n_1_. Gates G_1_, G_2_ and G_3_ are used to apply and control the electric field towards the bottom direction. A positive and negative voltage of 3.3 V and −1.0 V, respectively, are applied to gates G_1_ and G_2_ alternatively as high (H) and low (L) signal levels to modulate the photodiode. The gate G_3_ is maintained at a medium voltage of 1.5 V for the entire operation. The gate G_3_ is mainly used to control the created lateral electric field and the transfer and draining of charges. With a proper consideration of process parameters, device dimensions and careful selection of the doping concentration, a large electric field is created and a high-speed charge transfer is achieved. As a result, electrons converted from the incident photons are transferred to X and X’ direction by an alternate clocking of G_1_ and G_2_. A combination of eight LEFM sub-structure is utilized to build a large area LEFM, as shown in Figure 2b. The large area LEFM structure has a fully depleted light sensitive area of 32 μm × 32 μm and has all the identical inputs (i.e., gates G_1_, G_2_ and G_3_) and outputs of each sub-structure connected together in parallel by using a metal wire. The LEFM is implemented considering the size of the incident laser beam where the whole incident beam has to lay onto the large LEFM sensitive area. The proposed large LEFM structure is designed to achieve a higher output stability regardless of the fluctuation of the light beam spot location.

**Figure 3:**
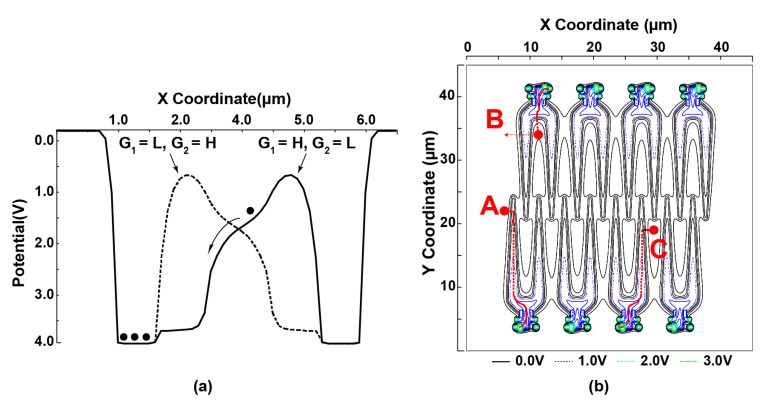
Two-step ion implantation large area Lateral electric field charge modulator (LEFM): (**a**) potential profile of sub-unit tow-steps ion implantation LEFM; and (**b**) photo-charges transfer time simulation from three different positions (A, B, and C).

To investigate the characteristics of the proposed two-step ion implantation large LEFM detector shown in Figure 2a, simulations using the SPECTRA device simulator have been conducted. The simulation of the potential profile along the X-X’ axis, mentioned in the LEFM sub-structure illustration in Figure 2a, is shown in Figure 3a. In this simulation, the voltage of the gate G_3_ is always set to 1.5 V. Figure 3a shows in black solid line the potential profile for photoelectron transfer to the left (i.e., X’-X direction) when the gates G_1_ and G_2_ are set to High (3.3 V) and Low voltage (−1 V), respectively. The black dashed line in Figure 3a shows the potential profile for photoelectron transfer to the right when the gates G_1_ and G_2_ are set to Low (−1 V) and High voltage (3.3 V), respectively. The layout, dimension and applied gate driving voltage difference between G_1_ and G_2_, which is set to be equal to 4.3 V (i.e., −1 V to 3.3 V), create a high electric field and a potential profile toward X coordinate direction attracting photoelectrons towards gates G_1_ and G_2_.

To ensure sufficient photo-charge transfer speed, a photo-charge transfer time simulation starting from three different charge positions (A, B and C) has been performed, as shown in Figure 3b, where the photo-charge path is also indicated by a red dotted line. Table 1 shows the simulation results of the photo-charges transfer time. The proposed detector structure can ensure a complete charges transfer of less than 4 ns, which makes it suitable for high-speed modulation applications and satisfy the requirement of the present application where the used demodulation clock is 20 MHz.

### 2.2. Lock-In Pixel, Readout Circuit and Their Operations

**Figure 4:**
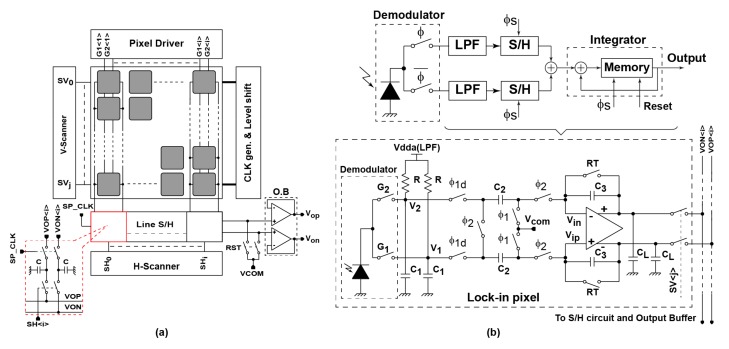
SRS lock-in pixel readout structure: (**a**) block diagram of the SRS CMOS imager; and (**b**) lock-in pixel readout circuit with block diagram and circuit implementation.

Figure 4a shows the block diagram of the proposed SRS lock-in pixel CMOS sensor with a 2-D pixel array. It includes a lock-in pixel array, a pixel driving circuit, a sample and hold circuit (S/H), a clock generation and level shifting circuit to generate the lock-in pixel clocking signals, a horizontal and vertical scanner and a pair of output buffers. Both the detector and the readout circuit are incorporated in the pixel. The SRS pixel includes the large area LEFM detector with two steps ion implantation, a pair of low-pass filters, a sampling circuit and an integrator using a fully differential operational amplifier (Op-Amp).

Figure 4b depicts the block diagram and the corresponding readout circuit structure of the lock-in pixel. The in-pixel readout circuit includes a pair of low-pass filter implemented with a 1st order RC filter with a separate power supply (vdda) for further reduction of power supply noise. Sampling and integration of the LPF differential output signal are performed using a sample and hold circuit (S/H) and a fully differential switched capacitor (SC) integrator.

Clocks G_1_ and G_2_ set the modulation frequency of the demodulator. Initially, the readout circuit integrator is reset by turning on the switches controlled by RT, φ_1_ and φ_2_. After resetting the integrator, the switches controlled by RT and φ_2_ are turned off and the switches controlled by φ_1_ and φ_1d_ with a brief delay are turned on, the pair of sampling capacitors C_2_ is then ready for sampling. The switches controlled by φ_1_ and φ_1d_ are turned off after the LPF outputs are sampled. Then, the differential charge stored in the two capacitors C_2_ is transferred to the capacitors C_3_ by turning on the switches controlled by φ_2_. This operation is repeated along the integration phase and the final SC integrator gain is given by the total number of cycles and the capacitor ratio of C_2_/C_3_. The operation is aimed to extract and amplify only the small SRS signal generated by the stimulated scattering process, which contains a large offset signal. To ensure a better performance, where a very weak SRS signal compared to the offset signal (i.e., 10^−5^~10^−6^) can be detected and extracted, low-frequency noise component (i.e., 1/f noise) introduced by the lock-in circuitry is needed to be removed. Removing 1/f noise component is performed using double modulation technique while the white noise is reduced by increasing the observation time and averaging [16].

## 3. Measurement Results and Discussion

### 3.1. Chip Implementation

**Figure 5:**
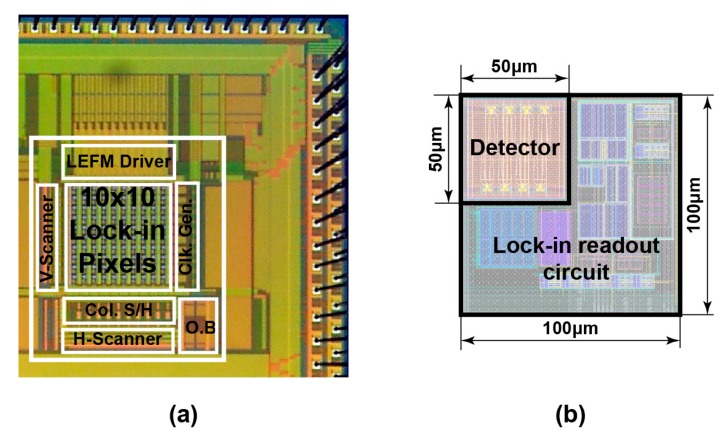
Micrograph of the SRS lock-in pixel CMOS imager prototype (O.B: output buffer; Clk. Gen: clock generator; LEFM: Lateral electric field modulator; S/H: sample and hold): (**a**) micrograph of the proposed SRS sensor chip; and (**b**) lock-in pixel layout for area sensor.

To characterize and experimentally evaluate the performance of the proposed SRS CMOS image sensor, a prototype chip has been manufactured using 0.11 μm CMOS image sensor process technology. Figure 5a shows the micrograph of the SRS sensor chip, which includes a 10 × 10-pixel array where the detector and the lock-in readout circuit are placed in each pixel with the area of 100 μm × 100 μm. The entire chip includes many test structures, but those are not shown in Figure 5a. Figure 5b depicts the SRS pixel layout of the detector and the lock-in readout circuit. In this preliminary work, and to demonstrate the operation of the proposed SRS CMOS sensor, only a single pixel is selected and characterized.

### 3.2. Experimental Setup

**Figure 6:**
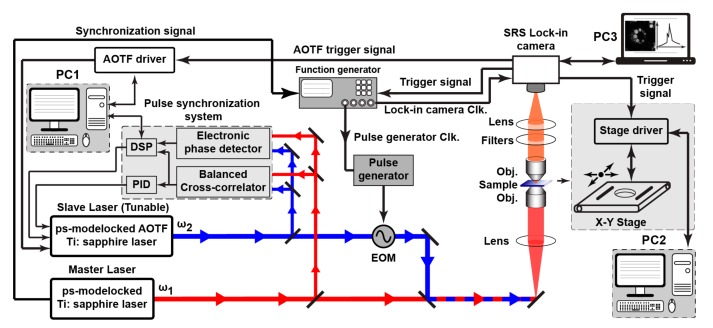
SRS experimental setup. AOTF: acousto-optic tunable filter; EOM: electro-optic modulator; Obj.: microscope objective; DSP: digital signal processing unit; PID: analog proportional-integral-differential.

The simplified schematic illustration of the proposed hardware experimental setup for SRS spectrum and imaging measurement system developed for bioimaging and used to characterize the proposed lock-in pixel of the SRS image sensor is depicted in Figure 6. Two laser sources are used in this setup. The master laser source consists of a picosecond mode-locked Ti:sapphire laser used to generate the unmodulated (DC) laser, also called the pump laser. Throughout the experiments and measurements, the pump laser light is running at 80 MHz and with a wavelength fixed at 709.06 nm. The second laser source, a tunable slave laser so-called the Stokes laser is used to generate the modulated (AC) laser. This laser source which consists of a picosecond mode-locked Ti:sapphire and equipped with an acousto-optic tunable filter (AOTF) inside the laser cavity for rapid wavelength tuning [20], is running at 80 MHz and the wavelength is initially fixed at 888 ns. The AOTF is tunable from 800 nm to 940 nm. Wavelength scanning required for the SRS spectrum measurement is ensured by the computer controlled AOTF through the AOTF driver. For automated wavelength scanning, the AOTF driver is triggered by the AOTF trigger signal generated by the SRS lock-in camera in accordance with spectrum measurement timing. The wavelength of the Stokes laser is changed at the rising edge of each trigger signal to cover a predefined wavelength range with a predefined stepping. The setup also includes a pulse synchronization system built using a balanced cross-correlator [20], an electronic phase detector, a digital signal processing unit (DSP) and an analog proportional-integral-differential (PID) controller in order to synchronize the two laser. For both the pump and the Stokes lasers, the typical pulse duration is in the order of 10 ps. The electro-optic modulator (EOM) shown in the experimental setup is used to modulate the Stokes laser at 20 MHz. The SRS lock-in camera is synchronized to lock-in camera clock signal generated by the function generator which is being synchronized to the master laser. The function generator synchronization signal is generated inside the master laser using a photodetector and a high-speed Emitter-Coupled Logic (ECL) counter. Since here only one SRS lock-in pixel is characterized, we use a computer controlled X-Y moving stage for imaging measurement. The employed X-Y scanner (i.e., Max201 from Thorlabs, Tokyo, Japan) is controlled by a bench stepper motor controller (BSC102) from the same manufacturer. In the SRS imaging, the movement of the X-Y scanner is fully automated and is synchronized to the rising edge of a trigger signal generated by the SRS lock-in camera.

### 3.3. Measurement Results and Discussion

**Figure 7:**
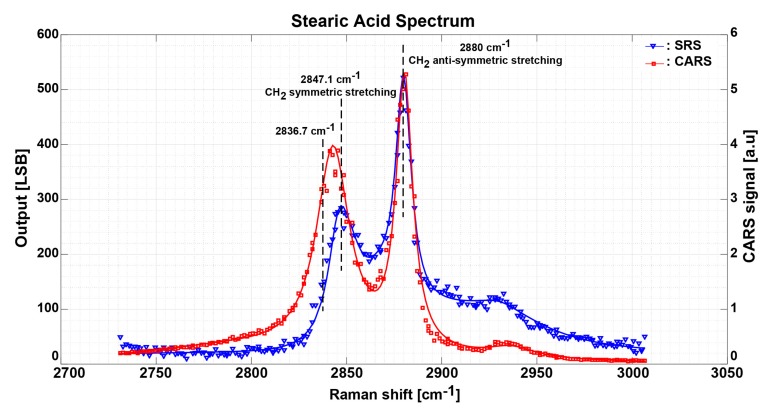
Measurement results of the stearic acid SRS spectrum in solid blue line and CARS spectrum in solid red line. Each SRS data point takes the average of 10,000 samples with total imaging time of 15 min.

Two samples are prepared, stearic acid and 3T3-L1 adipocyte cells. Initially, the power of the pump laser and the Stokes laser is set to 3.5 mW and 4.5 mW, respectively, and the total integration cycles are set to 700 cycles. The SRS spectrum measurements of these samples are performed after the sensor setup and calibration. As shown in Figure 7, the blue solid line is the measurement result of the SRS spectrum of the stearic acid sample by an automated scanning of the Stokes laser wavelength. The Stokes wavelength scanning is performed with 0.1 nm steps from 880 nm to 902 nm, which corresponds to 221 measurement points. The digital output code of the on-board 16-bit ADC is plotted as a function of the corresponding Raman shift and each data point of the spectrum is obtained by averaging of 5000 data points. The total measurement time, which includes triggering of the AOTF driver, wavelength changing, data readout, and saving, is around fifteen minutes. The CARS data for the same Stokes wavelength values are also recorded and plotted as a red solid line. The CARS is measured simultaneously with the SRS using a completely different system and detector (i.e., EMCCD) and is used for comparison and confirmation purposes. CH_2_ anti-symmetric stretching is observed at a peak of 2880 cm^−1^ for both SRS and CARS measurements and is in accordance with values presented in the different literatures [3,20,21,22,23] and in accordance to the Raman spectra [24,25]. On the other hand, SRS spectrum shows that CH_2_ symmetric stretching is observed at 2847.1 cm^−1^ while the CARS spectrum shows a little shift to the left at lower Raman shift. This is mainly due to the non-resonant background component of the CARS signal, which is dominant at lower Raman shift [26].

**Figure 8:**
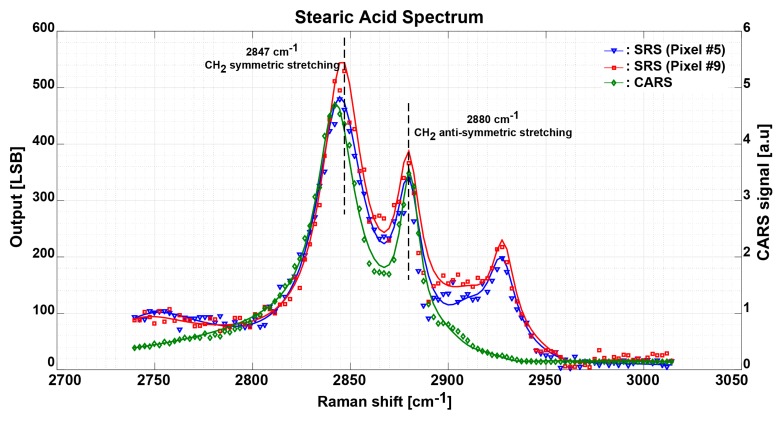
SRS spectrum of the stearic acid sample measured using Pixel #5 in blue solid line and Pixel #9 in red solid line, and CARS spectrum. Each SRS data point takes the average of 10,000 samples with total imaging time of 15 min.

To validate the operation of the proposed SRS image sensor, we also randomly measured the SRS spectrum of the solid state stearic acid sample using two other pixels different from the measurement in Figure 7. These two pixels are located in the first line column five and column nine of the 10 × 10 pixels array. For simplicity and ease of use, these two pixels have been named as pixel number five and pixel number nine (i.e., Pixel #5 and Pixel #9), respectively. In Figure 8, the blue solid line shows the SRS spectrum measurement result plotted as a function of Raman shift of the stearic acid sample using pixel #5 The SRS spectrum measurement result of the same sample using pixel #9 is plotted by a red solid line and measured CARS spectrum is shown by a green solid line. To speed-up the measurement time, the Stokes laser wavelength scanning is done with 0.2 nm step starting from 880 nm to 902 nm. In this measurement, CH_2_ anti-symmetric stretching is also observed at a Raman shift of 2880 cm^−1^ for both the CARS and SRS spectra of both pixels (i.e., Pixel #5 and Pixel #9). CH_2_ symmetric stretching is observed at a Raman shift of around 2847 cm^−1^ for SRS spectrum for both pixels while it is slightly smaller for the CARS spectrum due the non-resonant background of the CARS signal. Although the spectra shapes are different from the ones shown in Figure 7, CARS and SRS spectra show almost the same shape in Figure 8. CARS spectra shown in Figure 7 and Figure 8 are measured simultaneously with the SRS spectra using a completely different system and detector, and are used as reference spectra for SRS measurement. This is to prove that the proposed pixels are working and can be used for multi-focus SRS imaging later. Considering that the beam location was manually changed and both pixels were again calibrated before obtaining the spectra in Figure 8, the solid state stearic acid sample was exposed to laser irradiation for longer time. The sample may be heated up by irradiation which may lead to a possible change in the solid state stearic acid crystal structure and thus a change in the obtained SRS spectra. SRS spectra shown in the Figure 8 are similar to the liquid stearic acid spectrum in [25].

**Figure 9:**
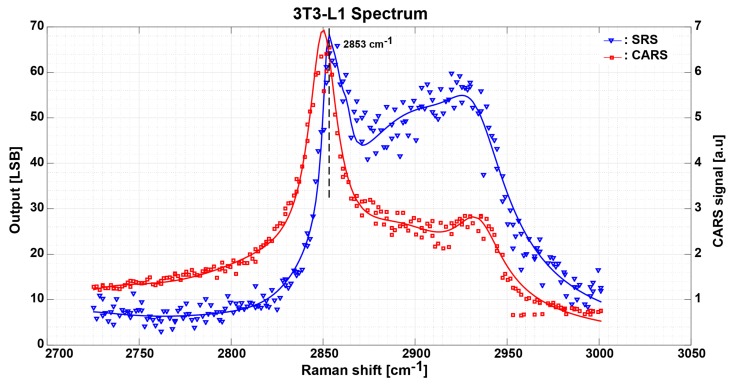
Measurement results of 3T3-L1 adipocyte cells SRS spectrum in solid blue line and CARS spectrum in solid red line. Each SRS data point takes the average of 10,000 samples with total imaging time of 15 min.

In Figure 9, the blue solid line shows the SRS spectrum measurement result of a lipid droplet in a 3T3-L1 adipocyte cell prepared on a slide glass. The measurement process for the 3T3-L1 adipocyte cell is the same as for the stearic acid sample. The red solid line as depicted in Figure 9 shows CARS data plotted for the same measurement points. CH_2_ symmetric stretching of lipid is clearly observed at 2853 cm^−1^ signifying the SRS spectrum for 3T3-L1 cells sample is successfully obtained. The spectrum is similar as reported in [5,7,8].

**Figure 10:**
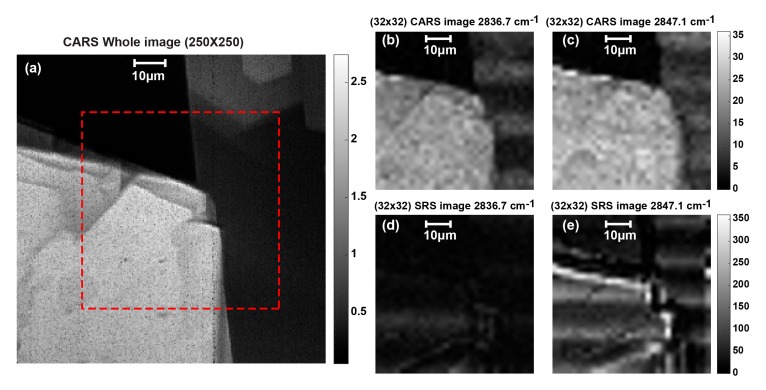
Stearic acid SRS imaging measurement taking the average of 10,000 samples with total imaging time of 75 min: (**a**) original 250 × 250 CARS image showing the scanning area in red dashed line; (**b**) 32 × 32 CARS image measured at 2836.7 cm^−1^; (**c**) 32 × 32 CARS image measured at 2847.1 cm^−1^; (**d**) off-resonance SRS image measured at 2836.7 cm^−1^; and (**e**) resonance SRS image measured at 2847.1 cm^−1^.

After performing the SRS spectrum measurement and confirming the Raman shift peak of each sample, a SRS imaging measurement is also performed for both biological samples. As a preliminary evaluation, a lower image resolution of 32 × 32 is taken with large X-Y scanning steps (i.e., 2 μm) in order to cover large imaging area. Since only one pixel is characterized, the total imaging time is estimated to be around 75 min including the X-Y stage moving, the total accumulation time and data saving to the measurement computer. For better visualization, the measured 32 × 32 images are resized and displayed as 255 × 256 images. Figure 10a shows the whole original 250 × 250 CARS image of the stearic acid sample with an approximate delimiter of the scanning area shown with the red dashed line.

Figure 10b,c shows the measured and resized CARS image of the stearic acid sample obtained by stage scanning at Raman shift of 2836.7 cm^−1^ and 2847.1 cm^−1^, respectively. Figure 10d,e shows the measured and resized SRS image of the stearic acid sample obtained by stage scanning at Raman shift of 2836.7 cm^−1^ and 2847.1 cm^−1^, respectively. Each of the reconstructed pixel SRS images is the average value of 5000 captured data. The stearic acid imaging shows that when the Raman shift is set to off-resonance (i.e., Raman shift = 2836.7 cm^−1^), the captured image is almost dark and no stimulated Raman signal is generated. On the other hand, when Raman shift is set to the corresponding resonance frequency of stearic acid sample (i.e., Raman shift = 2847.1 cm^−1^), stimulated excitation takes place and the SRS image is successfully observed. Comparison between Figure 10d,e prove that there is no stimulated Raman scattered signal when the Raman shift is set to off-resonance, even though CARS signal appears in Figure 10b because of the non-resonant background.

**Figure 11:**
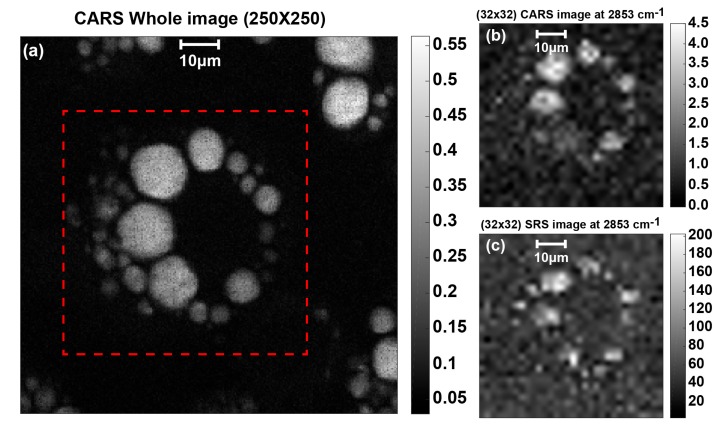
3T3-L1 SRS imaging measurement taking the average of 10,000 samples with total imaging time of 75 min: (**a**) whole 250 × 250 CARS image showing SRS scanning area in red dashed line; (**b**) 32 × 32 CARS image measured at 2853 cm^−1^; and (**c**) 32 × 32 SRS image measured at 2853 cm^−1^.

The SRS imaging of a lipid droplet in 3T3-L1 cells is performed at the CH_2_ symmetric stretching Raman shift (i.e., 2853 cm^−1^). Figure 11 shows the 3T3-L1 imaging results using the same parameters and scanning setup as for the stearic acid sample. Figure 11a shows the whole 250 × 250 CARS image with an approximate delimiter of the scanning area shown within the dashed red line. Figure 11b shows the measured and resized CARS image obtained by laser beam scanning. Figure 11c shows the measured and resized SRS image obtained by stage scanning. Each pixel of the reconstructed image is the average value of 5000 captured data. This figure shows that the SRS image of the lipid droplet is successfully obtained at the resonance Raman shift. For future works, SRS lock-in CMOS pixel array will be used in conjunction with multi-focus irradiation where less photo-damage is achieved [18,19]. Imaging time can be drastically improved and imaging at video rate using small CMOS image sensor could be achieved.

## 4. Outlook

Despite the relatively long time required for single point SRS spectra and SRS images measurement, the presented SRS lock-in CMOS pixel presents a good step toward the realization of real-time multi-focus SRS imaging with an SRS lock-in 2D-array image sensor working at video rate.

The reason for the long acquisition time of 75 min using single-pixel measurement for obtaining the images of Figure 10 and Figure 11 is explained as follows. The signal accumulation time of the SRS lock-in pixel including initialization time of 10 μs is 150 μs. In the current implementation, for better signal-to-noise ratio (SNR), the pixel output is sampled and read out 10,000 times and averaged over the samples and as a result the total time required is 1.5 s for one measurement positions. To obtain SRS images of Figure 10 and Figure 11, the current measurement system requires another 0.9 s for stage moving and 2.0 s for data writing to a storage. Therefore, the acquisition time for one measurement position is 4.4 s and the total required time to obtain images consisting of 32 × 32 measurement positions is about 75 min. The required lengthy imaging time is mainly due to hardware and software inefficiency since moving of the X-Y stage for sample scanning and data writing to storage is performed for every measurement positions with a single SRS pixel. The goal of this work is to achieve multipoint parallel measurement, where multi-focus excitation will be used on the pixel array to achieve high-speed imaging as well as reduction of photo-induced cell damage. With this goal, the time for stage moving and data writing is eliminated and the acquisition speed is determined by the signal accumulation, reading and averaging. When we implement a parallel measurement system using a *N_V_* × *N_H_*-pixel SRS image sensor and *N_V_* × *N_H_* multi-focus laser irradiation where *N_V_* and *N_H_* are vertical and horizontal pixels, the signal accumulation of 150 μs in all the pixels can be done in parallel and the total readout time for serially reading *N_V_* × *N_H_* pixels and the readout cycle time *T_P_* for one pixel is given by *N_V_* × *N_H_* × *T_P_*. The inverse of the readout cycle time per pixel, or 1/*T_P_* is called a pixel rate. Recent CMOS image sensors’ pixel rate is several 100 MHz to GHz. Therefore, the readout time can be very small compared to the accumulation time if *N_V_* and *N_H_* are small and readout circuits for high pixel rate is used. For instance, if we implement a 32 × 32-pixel SRS image sensor to obtain the images with the same resolution of Figure 10 and Figure 11, the total readout time is 10 μs with *T_P_* = 10 ns and the total acquisition time for one frame is 160 μs including the signal accumulation time of 150 μs. The total time required for taking 10,000 samples and averaging over the samples is 1.6 s.

**Figure 12:**
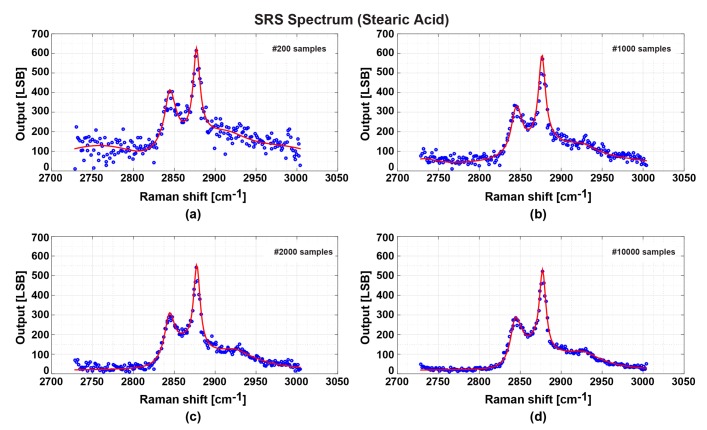
Comparison of the stearic acid SRS spectra obtained with different sampling numbers at each data point. Each point is the: (**a**) averaging of 200 captured data with measurement time of 30 ms; (**b**) averaging of 1000 captured data with measurement time of 150 ms; (**c**) averaging of 2000 captured data with measurement time of 300 ms; and (**d**) averaging of 10,000 captured data with measurement time of 1.5 s.

The spectra shown in Figure 7, Figure 8 and Figure 9 are the results after obtaining 10,000 samples and the averaging over the samples for better SNR. Figure 12 shows the SRS spectra of stearic acid using different number of samples. Through visual observation, The SNRs in Figure 12a–c are degraded compared with Figure 12d, but, if the degradation is tolerable, the time needed can be reduced to 30 ms for 200 samples, 150 ms for 1000 samples, and 300 ms for 2000 samples, respectively. The acquisition time with a 32 × 32-pixel SRS imager and multi-focus irradiation will be 30 ms with 200 samples for averaging if the readout time is neglected, and a real-time SRS imaging system with the frame rate of 33 Hz (=1/30 ms) is feasible.

For better SNR, improvements of pixel performance are necessary. Currently, the gain of the in-pixel integrator is 700 with the sampling frequency of 5 MHz. Efforts for increasing the gain of the integrator and sampling frequency will improve the SNR of the SRS imaging pixels at video rate. Figure 12a shows noisy signals at lower than 200 LSB. Considering the modulation frequency at 20 MHz, which suppresses the laser noise, the dominant noise appears to be the contribution from the circuit. Currently, the major noise comes from thermal noise (kT/C noise) of the capacitor C_2_ in the pixel circuit of Figure 4b. This noise can explain the noise level of the measured SRS spectrum of Figure 12. The value of C_2_ in the current design is 100 fF. The kT/C noise is calculated as follow

(1) $$V_{n}=\sqrt{\frac{kT}{C}}=\sqrt{\frac{1.38\times 10^{-23}\times 300}{100\times 10^{-15}}}\;=\;203.5\;\mu V_{rms}$$

This noise is accumulated in the in-pixel fully-differential integrator for intensifying the SRS signal. After 700 times of integration for the gain of 700 to the SRS signal, the appeared noise at the output of the integrator is given by

(2) $$V_{n,INT}=\sqrt{2\times 700}\;\times \;203.5\;=\;7.61\;mV_{rms}$$

which corresponds to 312LSBrms after 16-bit A/D conversion in the experiments. The factor of $\sqrt{2}$ in the above calculation is due to the fully differential circuit configuration.

The result of Figure 12a uses the averaging number of 200, and this reduces the noise by the factor of $1/\sqrt{200}$, and the resulting noise due to kT/C noise of C_2_ is 22LSBrms or 132LSBp-p. This noise can explain the noise level of Figure 12a.

Maintaining the same pixel pitch and extra effort of optimizing the layout, a value of four times large than the current value will be used. Another method is to increase the sampling rate from the current 5 MHz to 20 MHz. These two optimizations will improve the SNR to be similar to that of Figure 12c. Increasing the integrator gain can reduce the external readout time and the noise. However, increasing the amplifier gain requires changing the operational amplifier design. A gain-boosted operational amplifier is a good candidate, which can achieve an open-loop gain up to 120 dB but with a little increase of circuit complexity and dimension [27].

In single beam irradiation method, higher laser power is needed in order to achieve high-speed imaging. Considering the photo-damage to the biological sample is mainly induced by multiphoton events, which is proportional to the peak power of the incident laser, higher laser power increases the potential of greater photo-damage to the biological samples [18,19,28]. For single-focus and multi-focus systems obtaining signals at the same frame rate, multi-focus imaging requires longer exposure time but lesser peak laser power on each point. Such a technique reduces the induced photo-damage to the biological samples [18,19]. Conventional SRS imaging requires a separate high-frequency lock-in amplifier for each channel to demodulate the small SRS signal, which hinders parallel detection, as such system leads to higher hardware complexity and unrealistic cost. Moreover, CCD detectors suffer from low dynamic range, causing them unsuitable to be used for extraction of megahertz signal in SRS imaging. Currently, in the literature, parallel detection of SRS signals are limited by modulation frequency in the range of kilohertz, which exhibits substantial laser 1/f noise. The proposed 2-D CMOS lock-in pixel array that works in 20 MHz modulation frequency fills in the need of pixel array for SRS imaging. Another advantage of multi-point parallel detection is the use in hyperspectral detection, where a range of spectrum can be obtained in a single-exposure. Parallel detection is useful for obtaining the signal of a highly dynamic biological sample to avoid sample movement. Apart from the current work of the 2-D array, the authors are working on a 1-D array with slightly different pixel layout for parallel hyperspectral detection.

## 5. Conclusions

In this work, we have presented an SRS imaging technique using a new lock-in pixel design with a large-area LEFM and lock-in pixel readout circuits. The proposed lock-in pixel structure with a high-speed demodulation in combination with the double modulation technique has better detection performances by reducing the residual offset component, eliminating the mechanical noise due to the fluctuation of the laser beam and greatly reducing low-frequency noise components. The detection of small SRS signal in huge offset signal is successfully achieved. By selecting a single pixel, SRS spectra and images of stearic acid and 3T3-L1 adipocyte cells are successfully obtained. To demonstrate the performance of the proposed design and the possible uses for hyperspectral or multi-focus SRS measurement, SRS spectra are measured by different pixels in the imager array. At the current preliminary stage, the acquisition speed is restricted by two factors, the hardware limitation and the SNR. Further improvement in the pixel parameters to enhance SNR will enable video rate acquisition. These results are a step forward to achieve multi-point parallel detection of hyperspectral and multi-focus functionality, as well as miniaturized and very cost competitive SRS imaging system with less photo-damage to the biological samples.

## Figures and Tables

**Table 1:** Simulation results of photo-charges transfer time.

Initial Location	Transfer Time (ns)
A	3.825
B	0.4333.463
C	3.463
